# An Inactivated West Nile Virus Vaccine Candidate Based on the Lineage 2 Strain

**DOI:** 10.3390/vaccines12121398

**Published:** 2024-12-12

**Authors:** Mikhail F. Vorovitch, Ksenia K. Tuchynskaya, Yuriy A. Kruglov, Nikita S. Peunkov, Guzal F. Mostipanova, Ivan S. Kholodilov, Alla L. Ivanova, Maria P. Fedina, Larissa V. Gmyl, Evgeny S. Morozkin, German V. Roev, Lyudmila S. Karan, Galina G. Karganova

**Affiliations:** 1 Chumakov Federal Scientific Center for Research and Development of Immune-and-Biological Products of Russian Academy of Sciences (Institute of Poliomyelitis), Moscow 108819, Russia; vorovich_mf@chumakovs.su (M.F.V.); kruglov_ja@chumakovs.su (Y.A.K.); nikita.peunkov@yandex.ru (N.S.P.); moguzel@yandex.ru (G.F.M.); ivan-kholodilov@bk.ru (I.S.K.); ivanovaalla1967@mail.ru (A.L.I.); mariafedinamf@gmail.com (M.P.F.); lvgmyl@mail.ru (L.V.G.); karganova@bk.ru (G.G.K.); 2 Institute of Translational Medicine and Biotechnology, Sechenov First Moscow State Medical University, Moscow 119991, Russia; 3 Federal Budget Institute of Science «Central Research Institute of Epidemiology» of the Federal Service for Surveillance on Consumer Rights Protection and Human Wellbeing, Moscow 111123, Russia; morozkin@cmd.su (E.S.M.); roev@cmd.su (G.V.R.); 4 Research Center of Neurology, Moscow 125367, Russia; lskaran@mail.ru

**Keywords:** West Nile virus, West Nile fever, inactivated vaccine, immunogenicity, seroconversion, neutralizing antibodies, orthoflavivirus, protectivity

## Abstract

**Background**: West Nile virus (WNV) is a rapidly growing problem worldwide. The lack of emergency treatment and a safe licensed vaccine against WNV allows the virus to cause sporadic outbreaks of human disease, including fatal cases. Formalin-inactivated vaccines have been used for a long time and have been shown to be very safe and effective, especially in susceptible populations. **Methods:** By adapting tick-borne encephalitis vaccine production technology, we produced a laboratory-inactivated vaccine against WNV based on the strain SHUA, isolated from humans with a lethal WNV infection in the year 2021. **Results**: The potential vaccine was tested for safety in vitro and in vivo in outbred SHK mice of different ages, including PCR analysis of the brains of these mice to test for the absence of viral RNA after intracerebral injection. **Conclusions:** The inactivated whole-virion laboratory vaccine showed 100% seroconversion and immunogenicity against WNV strain SHUA-1, isolated from a lethal human case, and provided the mice with 100% protection from disease and death.

## 1. Introduction

*Orthoflavivirus nilense* (West Nile virus (WNV)) is a neurotropic orthoflavivirus that is transmitted mainly between mosquitoes and birds but which can also infect humans and other vertebrates. WNV is endemic worldwide, spanning Europe, Asia, Africa, Australia, and the Americas [1,2,3,4,5]. It induces West Nile fever (WNF) in humans and animals, especially in some species of birds and horses. While most WNV infections are asymptomatic in humans, approximately 1% of infected individuals experience severe central nervous system (CNS) lesions such as meningitis and encephalitis [4,6].

WNV is mainly transmitted by *Culex* mosquitoes, although some other species, including *Aedes albopictus*, *Anopheles maculipennis*, and *Ochlerotatus triseriatus*, can also serve as WNV vectors [7,8,9]. Human transmission of WNV has also been demonstrated through contact during medical procedures, transplacental transmission, and breastfeeding [6,9,10].

WNV is a member of the *Orthoflavivirus* genus (family *Flaviviridae*). Its genome is composed of a single-stranded, positive-sense RNA, with a size of around 11 kb. There is one open reading frame that encodes seven nonstructural (NS1, NS2A, NS2B, NS3, NS4A, NS4B, NS5) and three structural proteins (C, prM, E) [11,12]. The mature WNV virions have a spherical shape with a diameter of approximately 50 nm and include the structural proteins C, M/prM, and E.

WNV displays significant genetic diversity, with nine lineages currently identified [8,13,14,15,16]. Outbreaks of the disease in humans and animals are globally associated with lineages 1 and 2 [13,17,18,19,20,21,22], whereas WNV lineages 3–9 are found only in mosquitoes, ticks, birds, and amphibians in some regions [23]. In the last two decades, lineage 2 strains have been the predominant cause of WNV infection in Russia and Europe, replacing WNV lineage 1; however, co-circulation of the two lineages still occurs [24,25].

Since the late 1990s, there has been a rise in the occurrence of WNF in urban regions throughout Europe, the Middle East, and the United States. An increase has also been observed in both neuroinvasive cases and fatalities [8,26,27]. In the territory of the Russian Federation, localized foci with a sufficiently high risk of WNV infection currently exist in the Southern Federal District [16,28]. Additionally, an outbreak of WNV occurred in the Moscow region in 2021 [29].

The primary method for safeguarding individuals against various orthoflavivirus infections is through prophylactic vaccination. WNV vaccines for veterinary use have been licensed and are now commercially available. Two inactivated whole-virion vaccines, Innovator^®^ (USA) and Vetera^®^ WNV (Germany), show significant effectiveness; however, they require revaccination every six months for full animal protection [30,31]. A licensed recombinant live attenuated vaccine, Recombitek^®^ (Merial, Germany), was developed from recombinant live canarypox virus expressing prM and E WNV transgenes [30]. This vaccine has proven to be successful in testing for both dogs and cats. Additionally, there is a licensed commercial DNA vaccine available. Generally, licensed veterinary WNV vaccines have been developed using line 1 strains of WNV, a genotype of the virus that was distributed extensively throughout the United States and which caused outbreaks in Europe, the Middle East, and Russia during the mid-1990s [13,15,25].

Since 1999, significant research efforts have been expended to create WNV vaccines for disease prevention in humans. Currently, vaccines against WNV for humans under development include classical attenuated and inactivated vaccines, genetic vaccines, vaccines based on recombinant protein E, vector vaccines, and others undergoing preclinical and clinical studies [5,32]. These WNV vaccines, based primarily on lineage 1 strains, have shown promise in preclinical studies. However, their effectiveness varied, with some demonstrating limited immunogenicity against diverse strains of the virus, including lineage 2, which has emerged as an increasingly virulent strain [33,34,35,36,37]. Thus, at present, there is no licensed human WNV vaccine available.

One potential avenue for vaccine development is the use of a unified platform to create a range of vaccines. Previously, a vaccine against TBEV was developed using Vero cells (Evervac) [38]. Given that all orthoflaviruses exhibit similar physicochemical characteristics, a similar technology was employed to develop a vaccine candidate against WNV.

The objective of this work is to develop a laboratory version of the inactivated whole-virion vaccine for the prophylaxis of WNF in both animals and humans using the WNV genotype 2 topical strain, as well as the assessment of its immunogenicity and protectivity in vivo.

## 2. Materials and Methods

### 2.1. Cells

Pig embryo kidney (PEK) cells were cultured in a mixture of medium 199 in Hanks solution and medium 199 in Earle’s solution (2:1) (Chumakov FSC R&D IBP RAS (Institute of Poliomyelitis), Moscow, Russia) with 5% fetal bovine serum (FBS) (Invitrogen, Waltham, MA, USA) and antibiotics (100 U/mL penicillin, 100 μg/mL streptomycin) (Paneco, Moscow, Russia) at 37 °C. Vero cell culture (green African monkey kidney epithelial cells) was cultured in DMEM (Chumakov FSC R&D IBP RAS (Institute of Poliomyelitis), Moscow, Russia) supplemented with 2 mM L-glutamine, antibiotics (100 U/mL penicillin, 100 μg/mL streptomycin) (Paneco, Moscow, Russia), and 10% FBS (Gibco, Paisley, UK) at 37 °C. *Aedes albopictus* C6/36 cell culture was cultured in L-15 (Leibovitz) medium (Chumakov FSC R&D IBP RAS (Institute of Poliomyelitis), Moscow, Russia) with 2 mM L-glutamine, antibiotics (100 U/mL penicillin, 100 μg/mL streptomycin) (Paneco, Moscow, Russia), and 10% FBS (Gibco, Paisley, UK) at 28 °C.

### 2.2. Viruses

The poliovirus strain Sabin I was used as an internal control in the qRT-PCR [39]. WNV line 1 strain HP-94 isolated in the Astrakhan region, Russia, in 1963 from *Hyalomma marginatum* (GenBank number JX041633.1), which was used to obtain hyperimmune ascites fluid.

The SHUA strain of WNV utilized in this study was isolated from an individual diagnosed with West Nile neuroinvasive disease in the Moscow region during the summer of 2021 [29]. The complete genome of the virus from the patient’s blood was determined (GenBank number PP580188). The strain used to generate a vaccine candidate was WNV SHUA-3, which was isolated from the saliva of the same patient and passed through one passage in C6/36 cell culture and one passage in the brain of suckling white SHK mice aged 2 days. The WNV SHUA-1 strain was isolated from the serum of the same individual and underwent four passages in Vero cell culture, and it was used in experiments to evaluate the immunogenicity and protection of the vaccine.

### 2.3. Animals

BALB/c mice and outbred SHK mice (State Institution Scientific Center of Biotechnology, branch “Stolbovaya”, Moscow, Russia) were used. For virus isolation and to assess the completeness of inactivation of the virus-containing fluid (VCF) in vivo, 2–3-day-old sucklings of outbred white SHK mice and SHK mice weighing 7–8 g were employed. BALB/c mice weighing 16–18 g were used for experiments on the immunogenicity and protectivity of the vaccine candidate.

The mice were housed in an experimental animal facility in specialized rooms for work with pathogens of risk group III in accordance with the requirements for the conditions of laboratory animal care, including the European Council Directive 2010/63/EU on the protection of animals used for scientific purposes, the recommendations for the care and use of laboratory animals of the National Institutes of Health, USA, and SP 2. 2.1.1.3218-14 “Sanitary and epidemiological requirements for the design, equipment and maintenance of experimental-biological clinics (vivariums)”. All experiments with animals were conducted in compliance with ethical standards in accordance with CIOMS recommendations, 1985, Directive 2010/63/EC, Annex A to the European Convention ETS № 123, and on the conclusion of the Ethics Committee of Chumakov FSC R&D IBP RAS (Institute of Poliomyelitis), Moscow, Russia № 13032023 from 15 March 2023. Animals were euthanized when they showed obvious clinical signs of WNV infection. The health status of the animals was assessed at least once a day based on visual observation. The signs of the disease were considered to be ruffling, impaired locomotor activity, weakness of muscles of the fore and hind limbs, paresis, paralysis, and a weight loss of over 12% of the initial weight of the animal.

### 2.4. Titration of WNV

The virus titer was determined via the plaque method in Vero cell culture and expressed as the decimal logarithm of plaque-forming units per ml (log_10_ PFU/mL). Ten-fold serial dilutions of the virus in medium 199 were prepared using Earle’s solution. Next, 100 µL of the analyzed sample in 2 replicates was added to the Vero cell monolayer in 24-well cell plates (Corning, Germany). The plates were then incubated in a CO_2_ incubator at 37 °C for 1 h to facilitate virus adsorption. Following this, 1 mL of a coating solution consisting of 1.26% methylcellulose (Sigma, St. Louis, MO, USA) prepared in a mixture of 199 medium in Hank’s solution and 199 medium in Earle’s solution (in a 1:2 ratio) was added. The medium also contained 2% FBS (Invitrogen, Waltham, MA, USA) as well as a combination of streptomycin and penicillin antibiotics (Paneco, Russia). Following incubation for 6–7 days at 37 °C/5% CO_2_, the wells were washed twice with 199 medium in Earle’s solution. Cell fixation was performed with 96% ethanol for 1 h at room temperature under UV irradiation. Finally, cells were stained with a 0.4% solution of gentian violet dye (Alfa Aesar, Haverhill, MA, USA).

### 2.5. Production of Hyperimmune Ascites Fluids

Immune murine ascites fluid was obtained in SHK mice using a method previously described in [40]. Briefly, mice were immunized with a mixture of WNV strain HP-94 with complete Freund’s adjuvant three times subcutaneously (s/c) followed by a single immunization with the virus without injection of the adjuvant intraperitoneally (i/p). To obtain ascites, TG-180 sarcoma cells were injected i/p the day after the last immunization. Ascites and serum were collected on day 10–12 after the last injection.

### 2.6. Preparation of WNV-Containing Fluid

Two variants of cell culture cultivation were used:

1. The cultivation of monolayer culture was conducted in a 2 L roller bottle (TufRol, Thermo Fisher Scientific, Waltham, MA, USA) in cell production roller apparatus (BELLCO Biotechnology, Vineland, NJ, USA) in Vero cells at 37 °C;
2. Pseudo-suspension cultivation on Cytodex-3 type microcarrier beads (Cytiva, Marlborough, MA, USA) in 1.5 L spinner flasks at 37 °C. The seed concentration of Vero cells was 600–900 thousand cells/mL, and the concentration of Cytodex-3 microcarrier was 5–6 g/L.

Virus-containing fluid was obtained by infecting Vero cell culture with WNV strain SHUA-3; the multiplicity of infection was 0.1–0.5 plaque forming units (PFU) per cell. After infection, the virus was cultured in DMEM solution supplemented with 0.1% human serum albumin (HSA) (NPO “Microgen”, Moscow, Russia) and 50 μg/mL gentamicin (OAO “Dalchimfarm”, Khabarovsk, Russia). VCF was collected 48–72 h after infection once a mild visible cytopathic effect was observed.

### 2.7. Inactivation of Virus-Containing Fluid

To inactivate VCF, a 40% formaldehyde solution (AO “Retinoids”, Oryol, Russia) was added to a final concentration of 0.02%.

In vitro, the completeness of inactivation was determined by observing the absence of cytopathic effects on Vero cell monolayers. Cells were incubated for 7 days (37 °C/5% CO_2_) and observed microscopically daily.

To assess the completeness of inactivation of the VCF in vivo, inactivated samples were administered i/p and intracerebrally (i/c) in volumes of 0.5 mL and 0.03 mL, respectively, to 10 outbred white SHK mice 7–8 g and also i/c at a volume of 0.01 mL to 7 two-day-old sucklings of outbred white SHK mice. The animals were observed for 21 days. Daily evaluations included monitoring of weight changes, general condition, presence of clinical signs of the disease, and animal mortality. The brains of the suckling mice were also tested for the presence of viral RNA on the 14th and 21st days after the inactivated WNV injection. For RNA detection, reverse transcriptase quantitative real time PCR (RT-qPCR) was used.

### 2.8. Preparation of Purified Inactivated WNV

After inactivating VCF to remove cellular debris, we performed clarification filtration using a Fluorodyne II Kleenpak capsule filter (Pall Corporation, Port Washington, NY, USA) following the concentration on the Pellicon 2 Biomax cassette membrane with a 300K rating (Millipore, Temecula, CA, USA). The resulting concentrate was then collected and stored briefly at 2–8 °C or long-term at −60 to −70 °C before use. The inactivated WNV (iWNV) concentrate was further purified with gel filtration using Sepharose 6 FF as a sorbent (Cytiva, Marlborough, MA, USA). Flow spectrophotometry at a wavelength of 280 nm controlled the collection of chromatographic fractions. The purified iWNV antigen fraction was obtained. If necessary, a Pellicon XL Biomax cassette concentrator (Millipore, Billerica, MA, USA) was used to re-concentrate purified iWNV. During the purification processes, samples were collected. The concentration of iWNV was estimated using an enzyme-linked immunosorbent assay (ELISA) and Western Blotting and a standard sample of iWNV.

### 2.9. Determination of Total Protein

The total protein content was determined by using a Lowry protein assay [41]. The optical density was determined on a spectrophotometer at a wavelength of 405 nm. The protein concentration of the sample was determined from the bovine serum albumin (BSA) (Sigma-Aldrich, St. Louis, MO, USA) calibration curve.

### 2.10. Determination of Total DNA

DNA was extracted on K-sorb columns (Synthol, Moscow, Russia) according to the manufacturer’s instructions. A sample of genomic DNA from a transient culture of the Vero cell line was used as a control to construct a calibration plot, with a concentration of 1 µg/mL. The amount of DNA was determined with RT-PCR on the Rotor-Gene Q using a PCR-RV reagent kit Syntol R-412 (Synthol, Moscow, Russia) with Tag DNA polymerase.

### 2.11. Preparation of the Standard iWNV Sample

To obtain highly purified iWNV to use as the standard sample in ELISA, a solution of protamine sulfate (Sigma-Aldrich, St Louis, MO, USA) in TH buffer was added to the concentrated iWNV. The resulting solution was incubated for 60 min at 4 °C and then centrifuged for 30 min at 12,000 rpm at 4 °C (Beckman J2-21, Beckman Coulter, Brea, CA, USA). The supernatant was subsequently collected and centrifuged for 3 h at 25,000 rpm, 4 °C (Beckman-Coulter L90K, Beckman Coulter, Brea, CA, USA). The resulting sediment was resuspended in the TH buffer, layered onto a 15–60% sucrose density step gradient, and the gradient was centrifuged for 3 h at 35,000 rpm, 4 °C (Beckman-Coulter L90K, Beckman Coulter, Brea, CA, USA). The fractions of interest were determined using ELISA. The viral particles were resuspended in the TH buffer through centrifugation at 35,000 rpm for 2 h at 4 °C (Beckman-Coulter L90K, Beckman Coulter, Brea, CA, USA). The precipitate obtained was dissolved in the TH buffer, followed by the preparation of 50 µL aliquots, which were stored at −70 °C for future use.

### 2.12. Electrophoresis and Western Blotting

Protein electrophoresis was conducted under denaturing conditions on a 10% SDS-PAGE in the Mini-Protein Tetra System (Bio-Rad, Hercules, CA, USA). BSA (Combithek Boehringer, Mannheim, Germany), ranging from 0.5 to 2 μg, served as a standard to determine the E protein concentration. Proteins were stained in the gel using a 0.22% solution of Coomassie R-250 dye (Sigma, USA). The proteins’ molecular weight was estimated by using molecular weight markers of size 10–200 kDa (Thermo Fisher Scientific, USA). Subsequently, the gel was scanned through the GBOX-CHEMIXX6-E gel documentation system (SynGene, Iselin, NJ, USA). Band densitometry was then executed employing the ImageJ 1.52v program (NIH, Bethesda, MD, USA). Lastly, the sample’s E protein quantity was determined using a calibration curve.

After electrophoresis in 10% SDS-PAGE, the proteins were transferred onto a nitrocellulose membrane (Hybond ECL, Amersham, Piscataway, NJ, USA) with the use of a Mini Trans-Blot cell module (Bio-Rad, USA) for Western blotting. To block free binding sites, a combination of 5% skim milk (Semper, Sundbyberg, Sweden) and PBS-T buffer (80 mM Na_2_HPO_4_, 20 mM NaH_2_PO_4_, 100 mM NaCl, 0.1% (weight/volume) Tween-20) was applied. The membrane was incubated with detection antibodies using mouse polyclonal ascitic fluid to WNV at a dilution of 1:250 for 1 h at 37 °C. After washing, a commercial peroxidase conjugate against mouse IgG (Sigma, USA) was used. Incubation was continued for another hour at 37 °C, and washing was performed again. Finally, the formed immune complexes were detected using the ECL chemiluminescent reagent (Amersham, USA) and X-ray film (FujiFilm, Tokyo, Japan).

### 2.13. Enzyme-Linked Immunosorbent Assay

ELISA was conducted using the Bioskin-WNV AG kit (Bioservice, Berdsk, Russia), following the manufacturer’s protocol. Optical density (OD) values were measured at 450 nm with the Multiskan Sky Microplate Spectrophotometer (Thermo Fisher Scientific, Waltham, MA, USA). To estimate the E protein content, a calibration curve was established by using a standard iWNV sample, analogous to the calculations performed for TBEV, as previously reported. The built-in Thermo Scientific SkanIt PC 4.0 version software was employed for calculations.

### 2.14. Mouse Immune Serum After iWNV Immunization

BALB/c mice 16–18 g received two intramuscular immunizations 2 weeks apart of 50 μL of the WNV vaccine candidate. Fourteen days after the second immunization, whole blood was collected after decapitation. Serum was separated from clots via centrifugation at 1500× *g* and stored as aliquots at −20 °C.

### 2.15. Neutralization Assay

A monolayer of Vero cells in 24-well plates was used for the neutralization assay. The reaction included 4-fold dilutions of sera from immunized mice in medium 199 in Earle’s solution with 2% FBS (Invitrogen, Waltham, MA, USA). The sera’s dilutions were incubated for an hour at 37 °C with WNV samples diluted to a concentration of 30–40 PFU/well. After an hour, the serum/virus mixture (100 μL) was added to the wells of 24-well plates with Vero cells and incubated for another hour. Further procedures were identical to the titration procedure as described previously. Every experiment included controls, which consisted of the negative and positive mouse sera with known antibody titers. Neutralizing antibody titers (NAT) were calculated according to the modified method of Reed and Mench [42].

### 2.16. Control of Specific Safety of the Vaccine Candidate In Vivo

The purified iWNV was administered to 30 white 7–8 g SHK mice without sex differences. The vaccine, at a volume of 0.5 mL, was injected into the abdominal cavity and 0.03 mL was simultaneously injected into the brain. Observations were made for 21 days, and the presence or absence of clinical symptoms and animal deaths was noted.

To determine whether WNV genomic material was present in immunized mice following the vaccine injection, 26 BALB/c mice 16–18 g were separated into two equal groups. Group 1 received an i/c injection of 0.03 mL vaccine, while Group 2 received an i/p injection of 0.5 mL. The mice were monitored for 25 days. Mice from each group were euthanized at 1 h (N = 3), 10 (N = 5) and 25 (N = 5) days post injection. The brain was then extracted for further detection of the WNV RNA via qRT-PCR.

### 2.17. Titration of WNV in Mice

To determine the 50% lethal dose of the investigated WNV strain, BALB/c mice weighing 12–14 g were s/c infected with 100 µL of 10-fold virus dilutions. Clinical symptoms, including ruffled fur, hunched posture, limb paresis, paralysis, and weight loss were monitored for 21 days. The lethal dose of the virus that causes 50% mortality (expressed as log_10_ LD_50_/mL) was calculated using the Kerber method [43].

### 2.18. Evaluation of the Protectivity of the Vaccine Preparation

The protective efficacy of the vaccine preparation against WNV was assessed in BALB/c mice weighing 16–18 g. The mice received two s/c injections of the vaccine candidate in the withers, with a 14-day interval between doses, at a volume of 0.5 mL and doses of 36, 180, and 900 ng of E protein. After the second immunization, mice were i/p infected with WNV at a dose of 100 LD_50_ in a volume of 0.25 mL and monitored for 21 days post-infection. Clinical symptoms included ruffled fur, hunched posture, limb paresis, paralysis, and weight loss.

### 2.19. Reverse Transcription and Real-Time PCR

Reverse transcription and real-time PCR were utilized to determine the presence of RNA-containing particles in the sample. The viral RNA was extracted using TRI Reagent LS (Sigma, St. Louis, MO, USA) in accordance with the manufacturer’s protocol. Reverse transcription was performed using the WNRT-R primer (CGGTWYTGAGGGCTTACRTGG) and M-MLV reverse transcriptase (Eurogene, Moscow, Russia) following the manufacturer’s protocol.

PCR was carried out using WNRT-R (CGGTWYTGAGGGCTTACRTGG) and WNRT-F (CGGAAGTYGRGTAKACGGTGCTG) primers and probe-WNV as a probe ((FAM)-WCCCCAGGWGGACTG−MGB-(NFQ)) taken from [44] with modification (Table S1) on a DNA Engine Analyzer (Bio-Rad, USA) utilizing a real-time PCR kit (Syntol, Moscow, Russia) in accordance with the manufacturer’s instructions. To control RNA extraction and reverse transcription, the Sabin I strain of poliovirus was used as an internal positive control and added in equal concentration to each sample before extraction [39]. The qPCR primers utilized for the internal control comprised PVR1 and PVL1: 5′-CGAACGTGATCCTGAGTGTGTT-3′ and 5′-GGCAGACGAGAGAAATACCCAT-3′, respectively, while for the PVP probe 5′-(R6G)-TTGATTCATGAATTTCCTCCTTCATTGGCA-(BHQ1)-3′ was utilized.

### 2.20. Genome Sequencing of WNV Strains

To obtain the complete genome of WNV (WNV-SHUA-1, WNV-SHUA-3), we used the specific primers (Table S2). The PCR products obtained were analyzed in an agarose gel, and bands of the target length were extracted from the gel. The bands were purified using the QIAquick Gel Extraction Kit (QIAGEN, Hilden, Germany) and sequenced on the Applied Biosystems 3500 Genetic Analyzer (Waltham, MA, USA) using the BigDye Terminator v3.1 Cycle Sequencing Kit (Thermo Fisher Scientific, Vilnius, Lithuania). The resulting sequences were analyzed using Lasergene^®^ SeqMan Pro software version 7.0.0 (DNASTAR Inc., Madison, WI, USA).

### 2.21. Phylogenetic Analysis

The RNA sequences of some strains/isolates of West Nile virus and the strain described in this article were used in the phylogenetic analysis. The nucleotide sequences of the complete open reading frame (ORF) were aligned using ClustalW. To identify ORFs, we used the Snap Gene Viewer program with translation options: minimum length of 75 amino acids and selected the options “Require a start codon ATG”, “except at DNA ends” and “Standard” of the genetic code for ORFs and new features. The phylogenetic analysis was conducted using the maximum likelihood method based on the General Time Reversible model in MEGA X with 1000 bootstrap replications [45,46].

### 2.22. Statistical Analysis

Fisher’s exact test was used to determine the statistical significance of differences between animal groups with regard to the survival rate of mice, absence of clinical symptoms of WNF, and presence of virus RNA in the CNS. The Log-rank or Fisher’s exact test was employed to determine the statistical significance of differences in life expectancy and incubation period, utilizing GraphPad Prism 9 software.

## 3. Results

### 3.1. Vaccine Strain Selection

**Figure 1:**
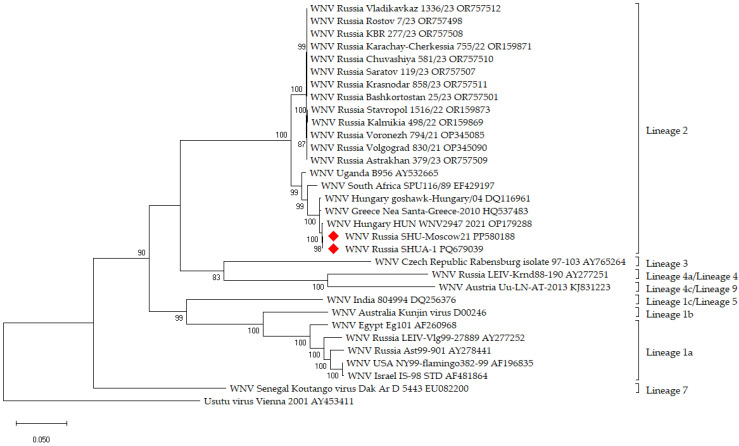
Phylogenetic positioning of isolate WNV SHU-Moscow 21 and strain SHUA-1 (marked with red rhombus) within the species WNV based on complete open reading frame. Maximum likelihood method based on the General Time Reversible model (1) of MEGA X with 1000-fold bootstrap analysis, rooted against the sequence of Usutu virus. The percentage of trees in which the associated taxa clustered together as shown next to the branches. The tree is drawn to scale, with branch lengths measured in the number of substitutions per site.

Viral RNA was extracted from the blood of a person diagnosed with neuroinvasive West Nile disease in the Moscow region in the summer of 2021. The whole genome sequence, obtained using high-throughput sequencing, showed that this isolate was grouped with Russian and European strains of WNV lineage 2 (Figure 1).

The strains SHUA-3 and SHUA-1 used in this work were isolated from the saliva and blood of the same individual, respectively. The SHUA-1 strain underwent four consecutive passages on Vero cell culture. The SHUA-3 strain underwent two consecutive passages on C6/36 cell culture and one passage on the brain of suckling mice.

After Sanger sequencing, the structural part of the genome sequences of two strains (SHUA-3 and SHUA-1) were compared with the isolate SHU-Moscow21. There were no nucleotide substitutions in complete open reading frame between isolate SHU-Moscow21 and strain SHUA-1, or in structural part of the genome in the isolate SHU-Moscow21 and strains SHUA-1 and SHUA-3. The SHUA-3 strain was chosen for further work after one passage in Vero cells.

### 3.2. Selection of Animal Models

**Figure 2:**
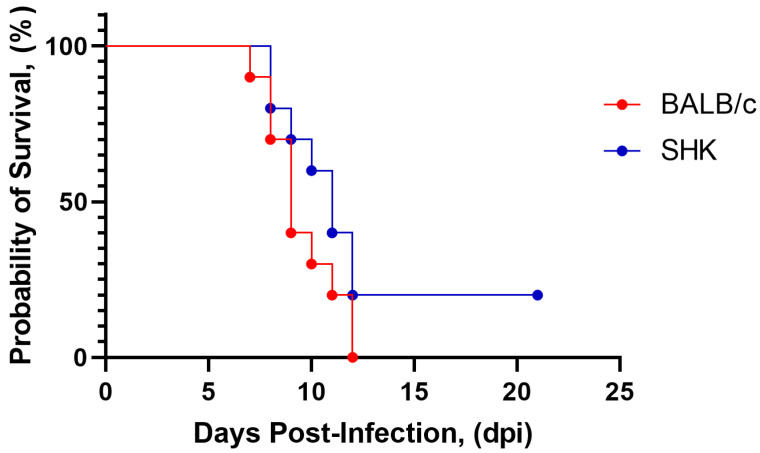
Survival of BALB/c and SHK mice following i/p infection of 10^7^ PFU per mouse SHUA-3 strain of WNV. N = 10.

Two lines of mice were selected as potential models for testing: inbred BALB/c mice (16–18 g) and non-linear (outbred) SHK white mice (7–8 g). To assess the susceptibility of mice to infection with strain SHUA-3, we i/p injected BALB/c and SHK mice with the virus at a dose of 10^7^ PFU per mouse. On the 12th day after injection, we observed 100% mortality in the BALB/c mice group and 80% mortality in the SHK mice group (Figure 2). There was no statistical difference between the curves determined with the Log-rank test (*p* = 0.13). As the mouse lines studied were susceptible to infection with the SHUA-3 strain of WNV, we used this mouse model to further evaluate the safety and efficacy of the vaccine candidate in vivo.

### 3.3. Inactivation of Viral Suspension

WNV was obtained in Vero cell culture as described previously [38]. Formaldehyde inactivation was performed at either 24 ± 1 °C or 32 ± 1 °C (Table 1). The E protein concentration in the analyzed samples of inactivated VCF was determined using the ELISA. First, the completeness of inactivation was tested based on the presence of CPE in Vero cells in vitro.

After 24 h of VCF inactivation at 24 °C, no cytopathic effect of WNV was observed in the Vero cell culture.

To assess the completeness of WNV inactivation at 24 °C for 24 h in vivo, we administered this VCF i/c and i/p to outbred white SHK mice weighing 7–8 g and intracerebrally to SHK suckling mice. SHK mice were observed for 21 days, and their survival rate was 100%, whereas in the control group, only 30% of mice survived (Table 2). In the case of intracerebral infection of suckling mice in the control group without administration of inactivated VCF, all mice (N = 7) died by seven dpi and all mice injected with inactivated VCF (N = 7) survived (Figure S1). Viral RNA was not detected in the brains of suckling mice after intracerebral injection of inactivated VCF on the 14th and 21st days, which is an additional confirmation of its safety.

Thus, based on the results of in vitro and in vivo experiments, our technique was effective in completely eliminating WNV infectivity, and the E protein concentration was slightly higher compared to other inactivation techniques. As a result, further inactivation of WNV in the VCF was carried out for 24 h at 24 + 1 °C.

### 3.4. Preparation of a Purified, Inactivated West Nile Virus (iWNV) Vaccine Candidate

Cellular debris was removed using cleaning filtration; VCF was concentrated through tangential ultrafiltration, and finally, gel filtration was performed using chemically modified agarose carriers.

A quantitative analysis of the West Nile virus E protein content was conducted using ELISA.

The total protein content in the iWNV vaccine candidate was less than 0.1 mg/mL. Moreover, analysis of the iWNV samples showed the absence of residual Vero cell DNA.

### 3.5. Safety of Purified iWNV Antigen In Vivo

To assess the safety of the purified iWNV antigen, it was administered simultaneously i/p (0.5 mL) and intracerebrally (0.03 mL) to outbred white mice. Three antigen doses were used: 900, 180, and 36 ng of E protein per mouse. The animals did not show any signs of illness after the administration (Table 3).

We also assessed the elimination of iWNV using the PCR assay after the above administration methods were performed. The brain was taken from the BALB/c mice weighing 16–18 g at various times after simultaneous intracerebral or i/p injection of the iWNV antigen at a dose of 180 ng of E protein per mouse. Viral RNA was detected in the brains one hour after intracerebral administration but was not detected on the 10th and 25th days (Table 4). In contrast, iWNV RNA was not detected in the brains of mice that received the i/p injection.

### 3.6. Immunogenicity and Protectivity of iWNV Antigen

**Figure 3:**
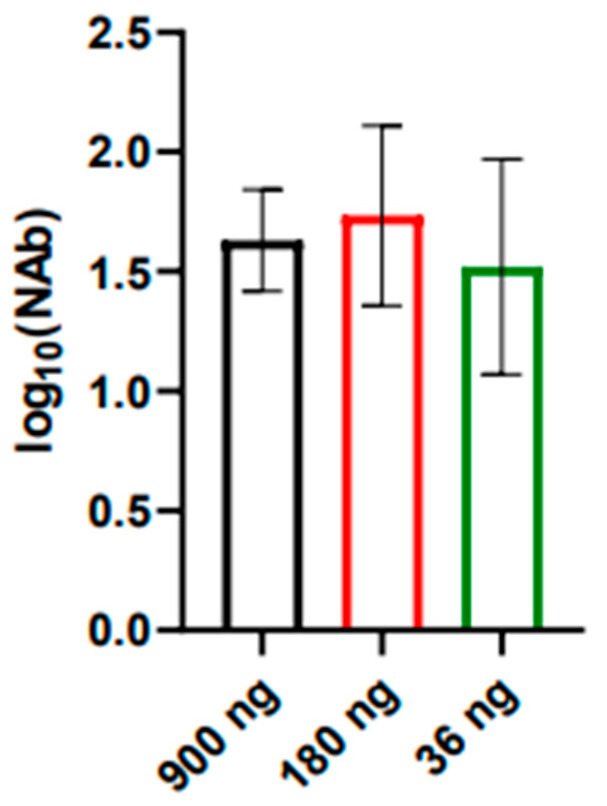
Neutralizing antibody (NAb) titers against the SHUA-1 strain of WNV two weeks after the last immunization with different doses of E protein. N = 3 for each dose in the group. Columns represent the mean values and error bars one SD.

The immunogenicity of the candidate vaccine was evaluated in BALB/c mice weighing 16–18 g. Samples at doses of 900, 180, and 36 ng of E protein per mouse were administered twice s/c in the withers (0.5 mL) with a 2-week interval between injections. At two weeks following the second immunization, blood was collected from each mouse, the sera were prepared, and the titer of virus-neutralizing antibodies against the strain SHUA-1 WNV was determined (Figure 3). Complete (100%) seroconversion was noted.

The obtained data indicate that the iWNV vaccine candidate demonstrates acceptable immunogenicity and induces greater than 1 log_10_ of neutralizing antibodies against the SHUA-1 strain of WNV in all immunized animals, even when administered at a dose of 36 ng protein E.

**Figure 4:**
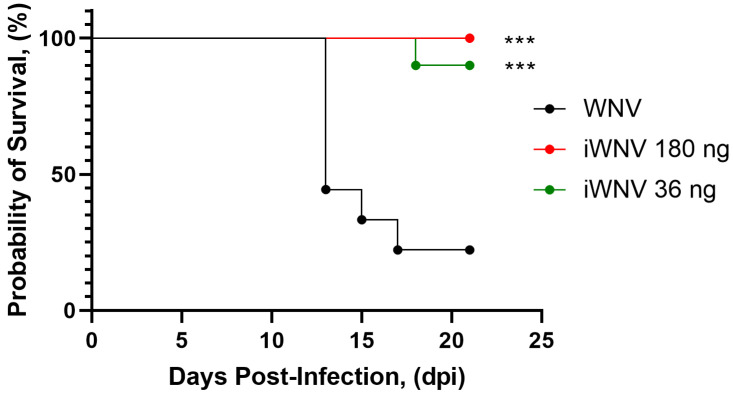
Survival of immunized mice following WNV infection with the SHUA-1 strain. ***—statistically significant difference between the experimental and control groups of mice (*p* < 0.0005) in Log-rank test. N = 10 in each group.

The protectivity of the vaccine candidate was evaluated in BALB/c mice. The mice were divided into three groups of ten animals each: a control group (without immunization) and two experimental groups. The vaccine was administrated s/c twice (0.5 mL), with an interval between injections of 2 weeks, in doses of 180 or 36 ng protein E. The control group received buffer solution, which was used to prepare the candidate vaccine samples. Fourteen days after the second immunization, mice were i/p injected with the SHUA-1 strain of WNV at a dose of 100LD_50_. Observation was carried out for 21 days after infection. The effectiveness of the vaccine was 100% at a dose of 180 ng and 90% at a dose of 36 ng (Figure 4, Table 5). Additionally, we evaluated the presence of WNV viral RNA using PCR in the brains of mice after WNV injection. No viral RNA was detected at 25 days post-infection in immunized mice, indicating that the immunized animals were effectively protected against the disease.

The laboratory variant of the inactivated whole-virion WNV vaccine demonstrates high protective activity in the investigated doses. Dose-dependent differences are not statistically significant, as determined using Fisher’s exact test.

## 4. Discussion

Modern reality constantly challenges us with outbreaks of new viruses or the emergence of new highly pathogenic variants of known viruses. The experience of the COVID-19 pandemic showed that having existing platforms for rapid response to viruses is the basis for combating them. One promising approach is to utilize already-existing platforms for vaccine production. Inactivated vaccines, being the safest, have a long history of use in human populations to protect against infectious diseases. They represent a promising solution of being able to contain new and re-emerging infections, especially in light of their safety when administered to children, adolescents, the elderly, cancer patients, and HIV-infected and other immunocompromised individuals. Like other arboviruses (Zika, chikungunya, and Mayaro), WNV demonstrates the capacity to increase its virulence while rapidly expanding its geographic scope [24,25,26]. It is evident that the list of arboviral infections that could spread in the future is likely to grow. It is therefore crucial to be prepared to respond quickly to any potential outbreaks.

At the present time, there appears to be no acute epidemiological necessity for a human WNV vaccine in Russia or globally. Nevertheless, the elderly, children, and immunocompromised patients continue to represent a risk group in endemic areas. As previously discussed, WNV has already demonstrated its capacity to disseminate rapidly to novel regions and precipitate substantial outbreaks with relatively high lethality. The utilization of a mature technology and a selected and well-tested inoculum virus can facilitate a rapid response to such a threat. The necessity for the extensive deployment of the vaccine will be determined by the epidemiological situation. Furthermore, such a vaccine could be employed not only for humans but also to safeguard birds and horses from outbreaks of WNV, which is highly lethal to them.

Inactivated whole-virion viral vaccines are chemically or physically modified virions [47,48]. Such vaccines are currently in use to prevent a number of flavivirus infections in humans, including Japanese encephalitis and tick-borne encephalitis vaccines [49,50]. New inactivated vaccines are also being developed to prevent yellow fever, dengue, Zika, and WNF [51,52,53,54].

Upon passages of WNV variants obtained from a patient with WNF, a number of viral variants were obtained. Given that the disease was fatal in this patient, it was deemed pertinent to utilize SHUA virus isolates to develop a laboratory-based candidate WNV vaccine. The SHUA-1 and SHUA-3 strains were obtained from this patient’s serum and saliva, respectively. Sequencing revealed that the nucleotide sequences of the structural regions of the SHUA-3 and SHUA-1 strains did not differ. The brain suspension of the SHUA-3 strain was utilized as a seed to accumulate WNV in Vero cell culture.

The selection of an animal model that allows the assessment of the safety and efficacy of a vaccine preparation in vivo is of paramount importance in the development of vaccines for the prevention of viral diseases. For a number of orthoflavivirus vaccines, mice are used as an animal model, as they are sensitive to infection with orthoflaviviruses and have a sufficiently high level of immunologic reactivity, both to assess humoral immunity and to evaluate the protective properties of vaccines [22,37,55,56]. Furthermore, the murine model is advantageous due to its low cost, ease of animal maintenance, and well-developed animal manipulation techniques, rendering it the optimal choice for large-scale production of orthoflavivirus vaccines [57,58]. However, different lines of mice exhibit varying degrees of susceptibility and sensitivity to different WNV strains [59,60]. The present study demonstrated that inbred BALB/c mice, non-linear (outbred) SHK mice, and 2-day-old suckling white SHK mice are susceptible to infection with the SHUA-1 and SHUA-3 strains of WNV. At both the intracerebral and i/p infection stages, the animals exhibited pronounced clinical symptoms, with mortality rates in the groups reaching 80–100%. Accordingly, these mouse lines were employed to assess the safety and protective efficacy of the vaccine candidate. In the future, these lines of mice can be utilized to develop methods for evaluating the safety and protective efficacy of the WNV vaccine during the commercial release of the drug.

A variety of chemical inactivators can be used to inactivate WNV [37,59]. In the present study, the traditional inactivator for orthoflaviviruses, formaldehyde solution, was used as the inactivating agent. Previously, we demonstrated that the use of formaldehyde as an inactivator for TBE virus under conditions of exposure to 32 °C for 72 h allows the preservation of most of the virion surface epitopes of E protein during the preparation of the inactivated TBE vaccine [61,62]. To inactivate WNV, we tested conditions that were less severe than those used for the TBE virus. As a result, WNV inactivation was performed at 24 °C for 24 h. Such inactivation conditions are expected to preserve the structure of most of the surface epitopes of the E protein, as observed in the case of the inactivated TBE vaccine. It was shown that the chosen inactivation conditions were sufficient to completely inactivate WNV; an absence of infectivity was observed in vitro and in vivo, including after intracerebral injection into two-day-old suckling SHK mice.

A methodology for the production of inactivated whole-virion TBE vaccines was previously developed at Chumakov FSC R&D IBP RAS (Institute of Poliomyelitis) [38,63]. This methodology subsequently served as the foundation for a platform for the development and production of several inactivated whole-virion vaccines, including hepatitis A vaccine, inactivated polyovaccine, inactivated YF vaccine, coronavirus vaccine Covivac, and a vaccine against hemorrhagic fever with renal syndrome [64,65,66,67,68,69]. The successful long-term experience of the practical application of inactivated whole-virion TBE vaccines prompted us to adopt the methodology of the TBE vaccine production as a basis for the development of a WNV vaccine candidate. The technological approach is distinguished by the following characteristics: the immediate inactivation of VCF upon receipt; the removal of cellular debris through clarifying filtration; the concentration of the vaccine by tangential ultrafiltration; and the use of chemically modified agarose as a carrier for gel filtration.

By employing this technological approach, we were able to obtain a candidate vaccine preparation based on the SHUA-3 strain. Additionally, the amount of purified WNV antigen, estimated from the concentration of protein E, can be set at a wide range of values. The maximum dose of antigen in the sample when using the aforementioned method of purified antigen preparation was 36 mg.

In subsequent experiments, the immunogenic properties of the candidate vaccine were evaluated against the SHUA-1 strain. Three antigen doses (36, 180, and 900 ng/mouse) were employed for the immunization of BALB/c mice. Intraperitoneal and intracerebral injection of these samples did not elicit any clinical symptoms in mice. It was demonstrated that immunization of mice with all three doses resulted in the effective induction of virus-neutralizing antibodies in 100% of the immunized animals. The protectivity rate was at least 80% even when immunized with a dose of 36 ng/mouse, and neutralizing antibody titers were about 1.5 log_10_. The data obtained for neutralizing antibody titers correlate well with the results obtained for WNV vaccines under development [48,70,71].

To assess the efficacy of the candidate vaccine against the strain SHUA-1, mice were immunized twice with the preparations at doses of 36 and 180 ng/mouse. The results demonstrated that all mice in the group that received the candidate vaccine at a dose of 180 ng/mouse survived, with no clinical symptoms of the disease and no detectable virus RNA in the brain on day 25 after administration of the 100LD_50_ dose of WNV.

## 5. Conclusions

A laboratory version of an inactivated whole-virion West Nile virus (WNV) vaccine candidate based on genotype 2 topical strain was developed in this study. The efficacy of “soft” conditions of West Nile virus (WNV) inactivation using formaldehyde solution was tested and demonstrated. The potential of using mice of the BALB/c and SHK lines for evaluating the safety, immunogenicity, and protectiveness of a WNV vaccine based on the SHUA-3 strain was also demonstrated.

## Figures and Tables

**Table 1:** WNV formaldehyde inactivation.

Indicators	Inactivation Temperature, °C	Initial VCF	Inactivated VCF		
			Time of Inactivation, hours		
			24	48	72
CPE	24 ± 1	+	-	-	-
	32 ± 1	+	-	-	-
E, ng/mL	24 ± 1	125 ± 12	80 ± 10	68 ± 4	63 ± 5
	32 ± 1	125 ± 8	70 ± 6	50 ± 4	53 ± 2

CPE—cytopathic effect: (+)—presence, (-)—absence. VCF—virus containing fluid.

**Table 2:** Evaluation of WNV inactivation completeness in vivo.

Inoculum	Survival of SHK Mice, 7–8 g, Simultaneous i/p and i/c Inoculation, N = 10, %	Survival of SHK Suckling Mice, i/c Inoculation, N = 7, %
WNV	30	0
iVCF	100	100 *

* All suckling mice died before 7 days of age. iVCF—inactivated virus containing fluid.

**Table 3:** Safety of the purified iWNV antigen in vivo.

E Protein Dose (ng per Mouse)	Route of iWNV Antigen Administration	Survival, N = 10, %
900	intraperitoneally (0.5 mL) and intracerebrally (0.03 mL)	100%
180		100%
36		100%

**Table 4:** WNV RNA elimination after intraperitoneal or intracerebral administration of 180 ng per mouse iWNV antigen determined using qRT-PCR in the brains of injected mice.

Route of Inoculation	Number of the Positive Samples (PCR) in the Mouse Brain/Number of Examined Mice	
Dpi, hours	i/c	i/p
1 h	3/3	0/3
10	0/5	0/5
25	0/5	0/5

dpi—days post-infection. i/p—intraperitoneal. i/c—intracerebral.

**Table 5:** Effectiveness of WNV vaccine candidate against SHUA-1 strain following double immunization of mice.

Antigen Dose, ng E Protein per Mouse	Mice, (Survival/Total)	Survival, %	Median Survival, Days	Mice Without Clinical Symptoms (Healthy/Total)	Mice Without WNV Viral RNA in the Brains, 25 Days After Infection (RNA Negative/Total) *
180	10/10 **	100	-	10/10	10/10
36	9/10 **	90	18	9/10	9/10
Control group	2/10	20	13 [11,12,13,14,15]	2/10	1/10

*—It was assumed in this study that WNV penetrated into the central nervous system (CNS) in deceased mice. **—statistically significant difference between the experimental and control groups of mice (*p* < 0.05; Fisher’s exact test).

## Data Availability

The data presented in this study are available in the article and Supplementary Materials. Obtained sequencing data were deposited in the GenBank database, West Nile virus, strain SHUA-1 (PQ679039), and isolate SHU-Moscow21 (PP580188).

## Supplementary Materials

The following supporting information can be downloaded at: https://www.mdpi.com/article/10.3390/vaccines12121398/s1, Figure S1: Evaluation of WNV VCF inactivation completeness in vivo on the SHK mice weighing 7–8 g; Table S1: Primers and probe for real-time PCR of West Nile virus. Table S2: Specific primers for amplification of whole genome of West Nile virus.
