# Supplementary material for: An Inactivated West Nile Virus Vaccine Candidate Based on the Lineage 2 Strain
Source: Vaccines (Basel). 2024 Dec 12;12(12):1398. doi: 10.3390/vaccines12121398 (PMC11680355; doi:10.3390/vaccines12121398)
Supplement: Supplementary file 1 [file vaccines-12-01398-s001.zip › vaccines-3334426-supplementary.pdf]

Supplementary Information

An inactivated West Nile virus vaccine candidate based on the lineage 2 strain

Mikhail F. Vorovitch [1,2], Ksenia K. Tuchynskaya [1*], Yuriy A. Kruglov [1], Nikita S. Peunkov [1], Guzal F. Mostipanova [1], Ivan S. Kholodilov [1], Alla L. Ivanova [1], Maria P. Fedina [1], Larissa V. Gmyl [1], Evgeny S. Morozkin [3], German V. Roev [3], Lyudmila S. Karan [4] and Galina G. Karganova [1,2]

1 Chumakov Federal Scientific Center for Research and Development of Immune-and-Biological Products of Russian Academy of Sciences (Institute of Poliomyelitis), Moscow 108819, Russia; vorovich_mf@chumakovs.su (M.F.V.); kruglov_ja@chumakovs.su (Y.A.K.); nikita.peunkov@yandex.ru (N.S.P.); moguzel@yandex.ru (G.F.M.); ivan-kholodilov@bk.ru (I.S.K.); ivanovaalla1967@mail.ru (A.L.I.); mariafedinamf@gmail.com (M.P.F.); lvgmyl@mail.ru (L.V.G.); karganova@bk.ru (G.G.K.)

2 Institute of Translational Medicine and Biotechnology, Sechenov First Moscow State Medical University, Moscow 119991, Russia

3 Federal Budget Institute of Science «Central Research Institute of Epidemiology» of the Federal Service for Surveillance on Consumer Rights Protection and Human Wellbeing, Moscow 111123, Russia; morozkin@cmd.su (E.S.M.); roev@cmd.su (G.V.R.)

4 Research Center of Neurology, Moscow 125367, Russia; lskaran@mail.ru

* Correspondence: kseniya-tuchka@mail.ru

Table S1. Primers and probe for real-time PCR of West Nile virus.

Name of primers	Nucleotide sequence	Primer direction	Temperature, ^{0}C
WNRT-F	CGGAAGTYGRGTAKACGGTGCTG	forward	60
WNRT-R	CGGTWYTGAGGGCTTACRTGG	reverse	
Probe_WNV	((FAM)-WCCCCAGGWGGACTG–MGB-(NFQ))	probe	

Table S2. Specific primers for amplification of whole genome of West Nile virus.

Name of primers	Nucleotide sequence	Usage	Primer direction	Genome locus	Amplicon size, bp	Temperature, ^{0}C
WNV-II-1f	TCGCCTGTGTGAGCTGACAAACT	PCR, Seq	forward	5′NTR – E	1736	58
WNV-II-2r	TCCTGCGACCCTAGAGCCACAA	PCR, Seq	reverse			
WNV_gE_4f	TCATTGGTTGGATGCTTGGAAG	Seq	forward	prM		
WNV-II-3f	CACGGTCAGGAATAGACACT	PCR, Seq	forward	E – NS1	1557	58
WNV-II-4r	AGGTGTCGTTGAGTCCGCTCT	PCR, Seq	reverse			
WNV-II-4f	TGGAGGAGCTTTTAGATCACT	PCR, Seq	forward	E – NS2a	1617	58
WNV-II-5r	ACGTCAGGCACTTCCCATGACA	PCR, Seq	reverse			
WNV-II-5f	CCGAGCATGGAACAGTATGGA	PCR, Seq	forward	NS1 – NS3	1799	58
WNV-II-6r	TGCCAAGCAGACCTCGAGTCAT	PCR, Seq	reverse			
WNV_g_13r	TTCTCCTGGTTGGTCCATCTCG	Seq	reverse	NS2a		
WNII-4370f	GACATGTGGATTGAGAGGACGGCT	PCR, Seq	forward	NS2b – NS3	1925	58
WNII-6290r	CGGTCATGGTATGATATTCCTGCTGCT	PCR, Seq	reverse			
WNV_Kgg_21f	GGTTTGTGCCCAGTGTGAAAATG	Seq	forward	NS3		
WNII-6185f	GGCTCAGAGGGGAAGAACGGAAGA	PCR, Seq	forward	NS3 – NS5	1655	58
WNII-7840r	GCCTCCAGTGATGTTTCCCTCT	PCR, Seq	reverse			
WNV_gE_25r	GGAACCATGTAGGCGTAGTGGC	Seq	reverse	NS4b		

WNII-7725f	GACTCAACCACATGACGAAGGAA	PCR, Seq	forward	NS5 – NS5	2134	58
WNII-9860r	CCGTCCTTCATGATCAGTTCCGT	PCR, Seq	reverse			
WNV_gE_31f	CAGCTCGCTGGTCAATGGAGTG	Seq	forward	NS5		
WNV_8966_R	CCTCACGCTCCTCATCCACC	Seq	reverse	NS5		
WNII-9790f	GGGTGGTATGACTGGCAGCAGGT	PCR, Seq	forward	NS5 – 3'NTR	1212	58
WNII-11010r	CGCACTGTGCCGTGTGGCTGGTTGT	PCR, Seq	reverse			
WNV_9936_R	ATCTGCGCGTATGACTTTG	Seq	reverse	NS5		

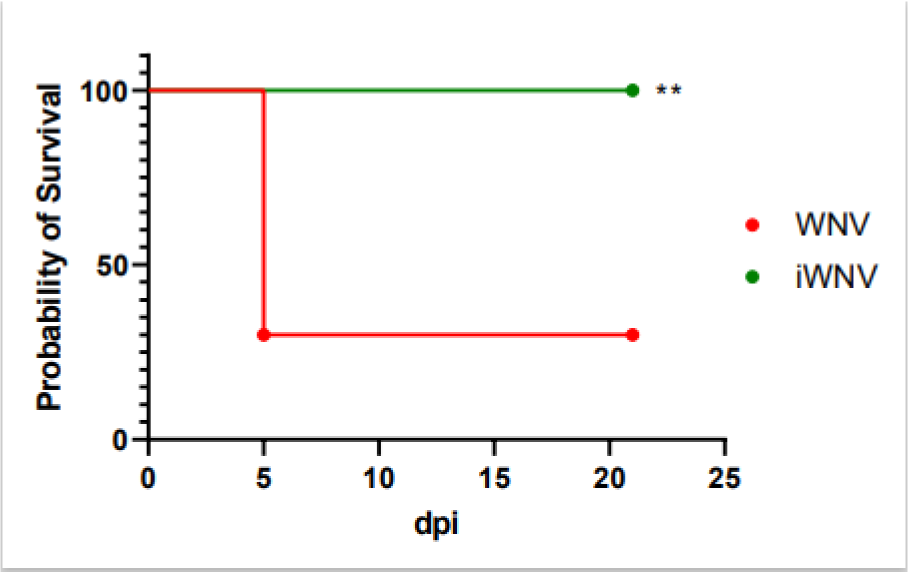

Figure S1. Evaluation of WNV VCF inactivation completeness *in vivo* on the SHK mice weighing 7-8 grams.

**statistical difference determined by Log-rank test
